# Spatiotemporal Pattern Analysis of Scarlet Fever Incidence in Beijing, China, 2005–2014

**DOI:** 10.3390/ijerph13010131

**Published:** 2016-01-15

**Authors:** Gehendra Mahara, Chao Wang, Da Huo, Qin Xu, Fangfang Huang, Lixin Tao, Jin Guo, Kai Cao, Liu Long, Jagadish K. Chhetri, Qi Gao, Wei Wang, Quanyi Wang, Xiuhua Guo

**Affiliations:** 1 Department of Epidemiology and Biostatistics, School of Public Health, Capital Medical University, Beijing 100069, China; gbmahara@gmail.com (G.M.); 13810147054@139.com (C.W.); xuqinhuachong@126.com (Q.X.); hff87hff87@163.com (F.H.); taolixin.2008@163.com (L.T.); guojin5827501@163.com (J.G.); anzhen602@163.com (K.C.); llstatistics@163.com (L.L.); gaoqi@ccmu.edu.cn (Q.G.); wei.wang@ecu.edu.au (W.W.); 2 Beijing Municipal Key Laboratory of Clinical Epidemiology, Beijing 100069, China; 3 Institute for Infectious Disease & Endemic Disease Control, Beijing Center for Disease Prevention & Control (CDC), Beijing Center for Preventive Medical Research, Beijing 100069, China; howardhuo@139.com; 4 Department of Geriatrics, XuanWu Hospital of Capital Medical University, Beijing 100069, China; chhetri_jk@hotmail.com; 5 Systems and Intervention Research Centre for Health, School of Medical Sciences, Edith Cowan University, Perth 6027, Australia

**Keywords:** scarlet fever, spatiotemporal patterns, children, Beijing

## Abstract

*Objective*: To probe the spatiotemporal patterns of the incidence of scarlet fever in Beijing, China, from 2005 to 2014. *Methods*: A spatiotemporal analysis was conducted at the district/county level in the Beijing region based on the reported cases of scarlet fever during the study period. Moran’s autocorrelation coefficient was used to examine the spatial autocorrelation of scarlet fever, whereas the Getis-Ord Gi* statistic was used to determine the hotspot incidence of scarlet fever. Likewise, the space-time scan statistic was used to detect the space-time clusters, including the relative risk of scarlet fever incidence across all settings. *Results*: A total of 26,860 scarlet fever cases were reported in Beijing during the study period (2005–2014). The average annual incidence of scarlet fever was 14.25 per 100,000 population (range, 6.76 to 32.03 per 100,000). The incidence among males was higher than that among females, and more than two-thirds of scarlet fever cases (83.8%) were among children 3–8 years old. The seasonal incidence peaks occurred from March to July. A higher relative risk area was mainly in the city and urban districts of Beijing. The most likely space-time clusters and secondary clusters were detected to be diversely distributed in every study year. *Conclusions*: The spatiotemporal patterns of scarlet fever were relatively unsteady in Beijing from 2005 to 2014. The at-risk population was mainly scattered in urban settings and dense districts with high population, indicating a positive relationship between population density and increased risk of scarlet fever exposure. Children under 15 years of age were the most susceptible to scarlet fever.

## 1. Introduction

Scarlet fever, also known as “scarlatina”, is a contagious disease caused by erythrogenic exotoxin, a substance produced by *Streptococcus pyogenes* (Group A Beta Haemolytic Streptococcus, GABHS)*,* which usually occurs in winter and spring and commonly affects children [1,2,3,4,5]. The incubation period ranges from 12 h to 7 days following contact with an infected person. Scarlet fever usually transfers through respiratory droplets (bacteria are spread through the nose) or direct contact with the mucus, saliva or skin of an infected person in the acute illness phase or sub-clinical stages [2]. However, it can also be transferred through food or contaminated milk [3,6]. The incidence of scarlet fever is high in overcrowded places, such as schools and other institutional settings. Although common among children 5–15 years of age, adults are also infected by the disease [7]. However, scarlet fever rarely occurs in children younger than 2 years of age because they are protected by maternal antibodies [8]. Notably, to date, there is no vaccine that prevents scarlet fever [9]. After treatment with antibiotics, scarlet fever can be completely cured and further complications can be prevented. However, if not treated in time, the disease can result in complications, such as septicaemia, vasculitis, hepatitis, glomerulonephritis and rheumatic fever, *etc.* [10,11,12].

Cases of scarlet fever have been documented since the early 18th century [13]. It became a common childhood illness in the 20th century. Today, scarlet fever rarely occurs in developed countries. However, it is a notifiable disease in developing countries, and outbreaks in teaching institutions have been recorded worldwide, including in Australia [14], Hong Kong [15], Taiwan [16], the United Kingdom and Mainland China [3,4,17,18]. In England, more than 1200 scarlet fever cases were reported from January to March in 2015 [19]. Thus far, scarlet fever has been continuously reported in China, the United Kingdom and Poland [19,20,21].

Several epidemiological studies of scarlet fever have been previously conducted in different parts of the world, for example, in Poland in 2012 [20], Hong Kong in 2011 [15,22], Northern Taiwan from 2001 to 2002 [23], Granada, Spain in 2012 [24], and in Beijing, Shanghai, and Hefei city in China in different years [3,25,26,27,28]. These studies have mainly focused on the molecular and epidemiological characteristics of the disease, as well as its antibiotic sensitivity [17,23,25,28,29]. However, a spatiotemporal model of scarlet fever seems to be lacking, and such a model might be helpful in determining high-risk areas during specific time periods. Therefore, a more location-specific public health intervention to control and prevent scarlet fever could be initiated [30,31]. The objective of this study was to identify the spatiotemporal patterns of scarlet fever at the district/county level based on the surveillance data gathered during our study period. Spatial, temporal, and spatiotemporal analyses were conducted to understand the trends and dynamics of transmission and to identify the high-risk areas and time frames of scarlet fever infection in Beijing, thereby providing reliable information concerning where and when prevention and control measures should be focused.

## 2. Methodology

### 2.1. Study Area

Beijing is the capital city of the People’s Republic of China. It is situated at the northern tip of the North China Plain, at 39°56’ N and 116°20′ E region, and it is surrounded by mountains to the north, northwest and west. The topography of the Beijing area slopes from the northwest to the southeast, and there is a continental climate characterized by hot, humid summers and cold, windy, and dry winters. Beijing measures 16,410.54 Sq km, with a population of 21.15 million. It consists of 16 districts (there were 18 districts before 2010) and 223 towns [32]. The districts are named as follows: (1) Dongcheng, (2) Xicheng, (3) Chaoyang, (4) Fengtai, (5) Shijingshan, (6) Haidian, (7) Fangshan, (8) Tongzhou, (9) Shunyi, (10) Changping, (11) Daxing, (12) Mentougou, (13) Huairou, (14) Pinggu, (15) Miyun, and (16) Yanqing [32].

### 2.2. Data Source

All reported cases (including the patient’s age, sex, occupation, and address) of scarlet fever from 1 January 2005 to 31 November 2014 were extracted from The Centre for Disease Prevention and Control China (China CDC) [5]. Additionally, we retrieved population data from the Scientific Data Sharing Center of Public Health, which was developed by the China CDC. During the study period, socio-demographic data from each district were obtained from the Beijing Bureau of Statistics of China. Maps of Beijing were downloaded from Data Sharing Infrasturucture of Earth System Science [33].

### 2.3. Case Definition

The diagnosis of scarlet fever is based on the clinical criteria established by the Law of Communicable Diseases Prevention and Control of The People’s Republic China and Guidance offered by the Chinese Ministry of Health. Scarlet fever is a notifiable Group B infectious disease, according to the China National Notifiable Infectious Disease Surveillance System (NNIDSS) [34]. The clinical manifestations of scarlet fever (ICD A38.01) are acute onset of fever, pharyngitis with strawberry tongue, red rash with a sandpaper feel, and itching, as well as a throat swab culture and stain and a skin smear stain to confirm *Streptococcus bacteria* (Group A) infection. By law, clinicians in China are required to report clinical cases of scarlet fever to the NNIDSS within 6 h after a diagnosis, and then the case will be added to the NNIDSS database [34].

#### 2.3.1. Ethical Approval

We obtained ethical approval from the ethical review committee of the School of Public Health, Capital Medical University (SPHCMU) Approval No. IRB00009511, Beijing, China. Consent from each individual subject was not required because we used only aggregated data from the CDC, Beijing, China.

#### 2.3.2. Human Participant Protection

This study was approved under the Institutional Review Committee for Healthcare Research and Quality protocol at Capital Medical University, Beijing, China, with secondary analysis of confidential data from the CDC, Beijing, China.

### 2.4. Statistical Analysis

All frequencies of scarlet fever were summarized annually, corresponding to each geographic area (e.g., district), and the incidence of scarlet fever per 100,000 population in each year was calculated by scarlet fever case counts, divided by that year’s population. Socio-demographic characteristics of scarlet fever patients in Beijing from 2005 to 2014 was calculated by using SPSS V-20 (SPSS Statistics 20, IBM: Corporation 1 New Orchard Road Armonk, New York, NY, 10504–1722, USA). Likewise, the address information and other data from each case were inputted into a Microsoft Excel 2010 spreadsheet (Microsoft: Redmond, WA, USA) and then linked to the corresponding location in ArcGIS software V 10.1 (ESRI, Inc., Redlands, CA, USA) [35]. For analysis, all collected data were managed in text format for support and recognition by the designed software.

#### 2.4.1. Spatial Autocorrelation Analysis

The spatial autocorrelation (Global Moran’s *I*) statistic measures were simultaneously based on the featured areas and values to analyse the degree of the dependency among the different observations in geographical space. Similarly, it also evaluates whether the disease patterns are clustered, dispersed or randomly distributed in the area [35]. In fact, the Global spatial autocorrelation statistic assesses the overall pattern and trend of the data, and it is used more extensively than other methods [36]. Thus, the autocorrelation statistic (Global Moran’s *I)* was used to detect the spatial autocorrelation of scarlet fever in the study locations by year. We used ArcGIS 10.1 software to compute the Moran’s *I* statistic.

In general, Moran’s *I* index values close to +1.0 indicate disease clustering, whereas *I* index values close to −1.0 indicate the dispersion of disease. Similarly, Moran’s *I* index value zero suggests that there is no disease clustering, indicating that the disease is randomly distributed in the study area [37,38].

We used local autocorrelation Moran’s *I* statistic to investigate the local level cluster (hotspot) locations of scarlet fever incidents. Anselin Local Moran’s *I* was used to determine whether there were positive spatial correlations (high-high clusters and low-low clusters) or negative spatial correlations (high-low clusters and low-high clusters). In fact, this statistic was used to detect the core clusters/outliers of districts with high scarlet fever incidences. Anselin spatial weights matrix is based on the inverse distance and weight between polygon centroids, which were constructed to identify the spatial relationships among the districts [39]. ArcGIS 10.1 was used to perform the analysis [40].

#### 2.4.2. Hot Spot Analysis

The local Getis-Ord statistic (Gi*), also known as hotspot analysis, was used to provide additional information that indicated the intensity and stability of core hotspot or cold spot clusters of scarlet fever in the entire study location [41,42,43,44,45]. The Getis-Ord Gi* statistic serves as an indicator for local autocorrelation, which measures how spatial autocorrelation varies locally over the study location and then calculates a statistic for each point data [46]. This method provides more intuitive results with a better visual exploration and also has the advantage of distinguishing high value clusters (hot spot) or low value clusters (cold spot). The degree of clustering and its statistical significance is estimated based on a confidence level, according to the Z-scores. If the Z (Gi*) score is positive and significant, it shows that one district and its neighbouring regions have a relatively high frequency of scarlet fever incidents, which is a hotspot (a spatial cluster of high data values); on the contrary, if the Z (Gi*) score is negative and significant, it indicates a cold-spot (a spatial cluster of low data values) [35]. Districts with Z-scores > 2.58 were considered to be significant at a 99% confidence level (*p* < 0.01), and they remained in the hot spot category. Districts with a Z-score between 1.65–1.96 and 1.96–2.58 were considered to be significant at 90% and 95% confidence levels (*p* < 0.10, and 0.05), and they were categorized as high risk districts. Whereas, a Z-score < −2.58 indicates the clustering of low values, indicating a cold spot [47]. The Gi* index was calculated as described in the previous study [41]. The output of the Gi* statistic presented on a map indicated the location of the spatial clusters in the study area, with the help of geographic information system (GIS) (ArcGIS, V-10.1).

#### 2.4.3. Retrospective SaTScan Analysis

SaTScan™ Software version-9.1 using the Kulldorff method of retrospective space-time scan statistic, was used to detect the geographical clusters of scarlet fever at the district/county level based on a discrete Poisson model. The space-time scan statistic is defined by a cylindrical window, with a circular geographic base and with height corresponding to the time period of possible clusters [48,49]. The base is centred around one of the several possible centroids placed in the study area, with the radius of the base varying continuously, according to the population range of the area, from zero to the maximum cluster size of the total population who might be at risk [50,51]. The window is then moved in space and time; therefore, it is possible to check all geographic locations and sizes, as well as each possible time period [52]. As a result, we obtained an infinite number of overlapping cylinders of windows of various sizes and shapes, jointly covering the entire study region, in which each cylinder reflects a possible cluster [49]. Thus, we performed the cluster analysis with the following default setting: maximum spatial cluster size, 50% of the population. The statistical significance of each cluster in the study area was based on comparing the likelihood ratio (*LLR*) against a null distribution achieved from the Monte Carlo simulation, with the number of replications set to 999 to ensure the sufficient statistical power. Additionally, the window with the maximum *LLR* is assumed to be the most likely cluster, that is, the cluster least likely to be caused by chance, and other windows with a statistically significant *LLR* were measured as secondary clusters [53].

In the likelihood ratio test, for each location and size of the scanning window, the alternative hypothesis is that there is an elevated rise within the window compared to outside the window. Under the Poisson assumption, the likelihood function for a specific window is proportional to Equation (1):

(1) $${\left(\frac{c}{E\left[c\right]}\right)}^{c}{\left(\frac{C-c}{C-E\left[c\right]}\right)}^{C-c}I\left(\;\right)$$

where “*C*” is the total number of cases, “*c*” is the observed number of cases within the window and “*E[c]*” is the covariate adjusted expected number of cases within the window under the null hypothesis. Because the analysis is conditioned on the total number of cases observed, “*C−E[c]*” is the expected number of cases outside the window. “*I*()” is an indicator function. When SaTScan is set to scan only for clusters with high rates, “*I*()” is equal to 1 when the window has more cases than expected under the null-hypothesis, and it is equal to zero otherwise. The opposite is true when the SaTScan is set to scan only for clusters with low rates. When the program scans for clusters with either high or low rates, then *I*() = 1 for all windows [53,54]. The scan window with the larger *LLR* value is defined as the most likely cluster; other scan windows with the *LLR* values with statistical significance are defined as secondary clusters. The relative risk (RR) of the incidence inside and outside the window is considered to be statistically significant if *p* < 0.05, which is evaluated by a Monte Carlo simulation [54,55].

In this study, the spatial units of space-time scan analysis were the 16 districts of Beijing, and the time units were 10 years (from 2005 to 2014). The time frame of the scan analysis was set to be one month to control for the time trends and to observe the cluster changes in the entire study period [54,55].

## 3. Results

### 3.1. Demographic Characteristics

A total of 27,688 scarlet fever cases were reported in Beijing from January 2005 to November 2014. We excluded 788 of these cases because the patients did not reside in Beijing, and we did not include 40 additional cases due to unavailable resident information; a total of 26,860 cases were included in the final analyses. The annual average incidence rate of scarlet fever was 14.25 per 100,000 populations (range, 6.76/100,000 in 2009 to 32.03/100,000 in 2011) (Table 1).

**Table 1:** Incidence of scarlet fever in Beijing from 2005 to 2014.

Year	No. of Cases	Incidence Rate (/10^5^)
2005	2164	14.7
2006	2268	14.16
2007	2392	14.27
2008	1798	10.15
2009	1258	6.76
2010	1665	8.50
2011	6466	32.03
2012	3367	16.27
2013	2170	10.26
2014	3321	15.43
Total	26,860	14.25 (Average)

Table 2 summarizes the sociodemographic characteristics of scarlet fever cases from 2005 to 2014. The reported cases of scarlet fever varied during the study period. The number was high in 2011 (6466 cases), followed by 2012 (3367 cases). The lowest number of cases was observed in 2009 (1258 cases). Among the total 26,860 cases, 63.2% (16,972) were male and 36.8% (9888) were female, with an average male-female sex ratio of 1.71:1. Most scarlet fever patients were 3–8 years old (83.8%, 22,511), and 9–15-year-old patients accounted for 10.3% (2757) of the cases over the study period. Children, who were spending their daytime at kindergarten or school, accounted for 40% (10,893), with primary level and secondary level students in the majority (48.7%, 13,074). Likewise, the morbidity rate of scarlet fever cases was higher in urban areas (62.2%, 16,716) than in rural areas (37.8%, 10,144) (Table 2).

### 3.2. Spatial Pattern

**Figure 1:**
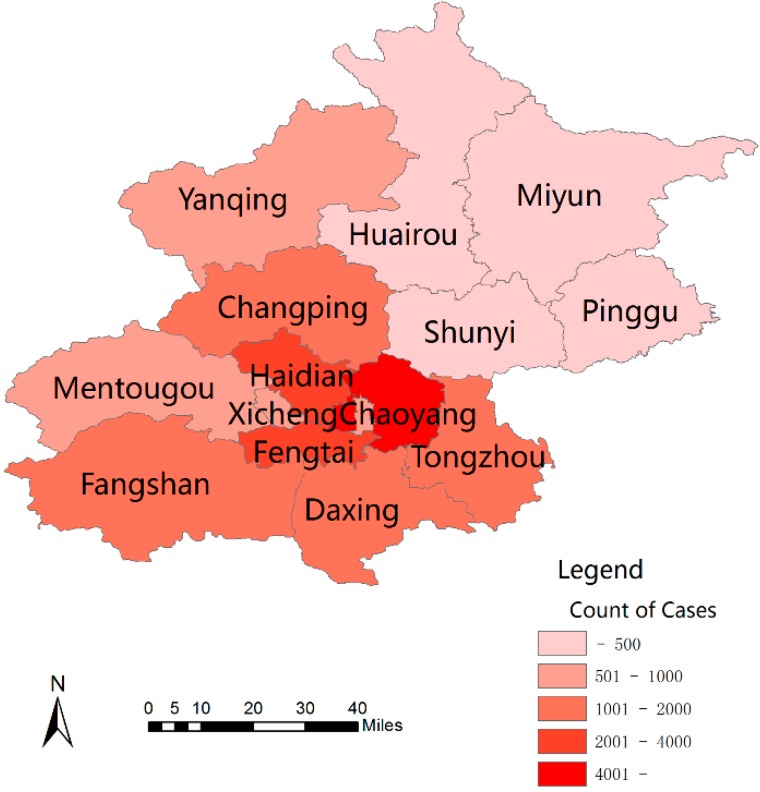
The distribution of scarlet fever cases in different districts in Beijing, 2005–2014.

The distribution of scarlet fever varied at the district/county level in the Beijing region during the study period (Figure 1). A relatively high incidence appeared in the districts of *Chaoyang* and *Xicheng*, and a slightly lower incidence was observed in the Haiden and Fengtai districts. However, a lower incidence of scarlet fever was observed in Changping, Fangshan, Tongzhou and Daxing districts across the entire study period (Figure 1).

**Table 2:** Socio-demographic characteristics of scarlet fever patients in Beijing, 2005–2014.

Group	2005	2006	2007	2008	2009	2010	2011	2012	2013	2014	Total
Gender	**–**										
Male	1358 (62.8)	1419 (62.6)	1523 (63.7)	1111 (62.1)	766 (60.9)	1050 (63.1)	4158 (64.3)	2173 (64.5)	1350 (62.2)	2064 (62.1)	16,972 (63.2)
Female	806 (37.2)	849 (37.4)	869 (36.3)	678 (37.9)	492 (39.1)	615 (36.9)	2308 (35.7)	1194 (35.5)	820 (37.8)	1257 (37.9)	9888 (36.8)
Sex Ratio	1.68	1.67	1.75	1.64	1.55	1.71	1.76	1.82	1.64	1.64	1.71
Age group	**–**										
Younger than 2 years	48 (2.2)	100 (4.4)	115 (4.8)	58(3.2)	40 (3.2)	81 (4.9)	314 (4.9)	163 (4.8)	82 (3.8)	152 (4.6)	1153 (4.3)
3–8 years	1981 (91.5)	1987 (87.6)	2041 (85.3)	1545(86.3)	1101 (87.5)	1448 (87.0)	5357 (82.8)	2637 (78.3)	1675 (77.2)	2739 (82.5)	22,511 (83.8)
9–15 years	123 (5.7)	167 (7.4)	215 (9.0)	163(9.1)	112 (8.9)	129 (7.7)	668 (10.3)	463 (13.8)	350 (16.1)	367 (11.1)	2757 (10.3)
Older than 16 years	12 (0.6)	14 (0.6)	21 (0.9)	23(1.3)	5 (0.4)	7 (0.4)	127 (2.0)	104 (3.1)	63 (2.9)	63 (1.9)	439 (1.6)
Districts	**–**										
Urban	1803 (83.3)	1950 (86.0)	1956 (85.7)	1533(85.7)	1076 (85.7)	958 (57.5)	3460 (53.5)	1896 (56.3)	1117 (51.5)	965 (29.1)	16,716 (62.2)
Rural	361 (16.7)	318 (14.0)	436 (18.2)	256(14.3)	180 (14.3)	707 (42.5)	3006 (46.5)	1471 (43.7)	1053 (48.5)	2356 (70.9)	10,144 (37.8)
Occupation	**–**										
Kindergarten Children	975 (45.1)	975 (43.0)	1024 (42.8)	919(51.1)	518 (41.2)	840 (50.5)	2595 (40.1)	1175 (34.9)	669 (30.8)	1173 (35.3)	10863 (40.4)
Scattered children	150 (6.9)	274 (12.1)	267 (11.2)	164(9.2)	107 (8.5)	218 (13.1)	673 (10.4)	310 (9.2)	190 (8.8)	397 (12.0)	2750 (10.2)
Student	1036 (47.9)	1015 (44.8)	1097 (45.9)	696(38.9)	632 (50.2)	604 (36.3)	3142 (48.6)	1846 (54.8)	1280 (59.0)	1726 (52.0)	13,074 (48.7)
Others	3 (0.1)	4 (0.2)	4 (0.2)	10(0.6)	1 (0.1)	3 (0.2)	56 (0.9)	36 (1.1)	31 (1.4)	25 (0.8)	173 (0.6)

Student (above than pre-primary level) and others (teachers, staffs of school). The bracketed figures indicate the percentage of cases in the corresponding year and group, and the urban districts included Haidian, Shijingshan, Fengtai, Chaoyang, Dongcheng, and Xicheng; the rural districts included Changping, Mentougou, Fanshan, Daxing, Tongzhou, Shunyi, Yanqing, Huairou, Miyun, and Pinggu for further analysis.

### 3.3. Temporal Pattern

**Figure 2:**
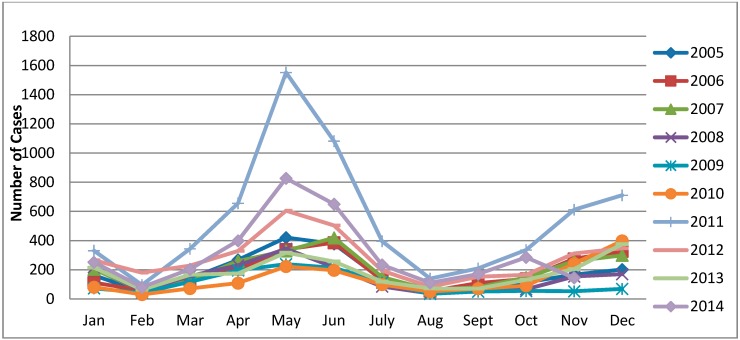
Monthly incidence of scarlet fever in Beijing, China, 2005–2014.

The monthly distribution of scarlet fever cases had significant seasonality and periodicity in Beijing (Figure 2). The first large seasonal peak occurred between March (early spring) and July (early summer), followed by a small pick from October (mid-autumn) to January (mid-winter). The highest incidence peak was observed in May of 2011, which was an epidemic year of scarlet fever in Beijing.

### 3.4. Spatial Autocorrelation Analysis

Table 3 describes the result of spatial autocorrelation (Global Moran’s *I*) analysis in Beijing. The value of Global Moran’s *I* increased from −0.004 to 0.141, and the Z score peaked (0.629 to 2.063) between 2005 and 2014; consequently, the distribution of scarlet fever was not spatially auto-correlated, indicating that the scarlet fever incidence was distributed randomly over that time period in Beijing.

**Table 3:** The results of the global spatial autocorrelation test of scarlet fever cases in Beijing, 2005–2014.

Years	Moran *I*	Z-Score	*p*-Value
2005	0.059	1.218	0.223
2006	−0.004	0.629	0.529
2007	0.053	1.228	0.220
2008	0.100	1.703	0.088
2009	0.141	2.063	0.039
2010	0.095	1.641	0.101
2011	0.064	1.412	0.158
2012	0.040	1.142	0.253
2013	0.040	1.172	0.241
2014	0.019	0.946	0.344

Anselin Local Moran’s *I* spatial analysis of scarlet fever incidence detected diverse patterns of spatial association from 2005 to 2014. This analysis shows that the strengthened high-high positive spatial association of scarlet fever incidence was in Fengtai district in 2005, which is an urban district in Beijing, and it also identified a high-high positive association in Chaoyang, Xcheng, Dongcheng and Fengtai districts in 2009; these areas are also urban districts, as well as the most densely populated districts of Beijing (Figure S1).

**Figure 3:**
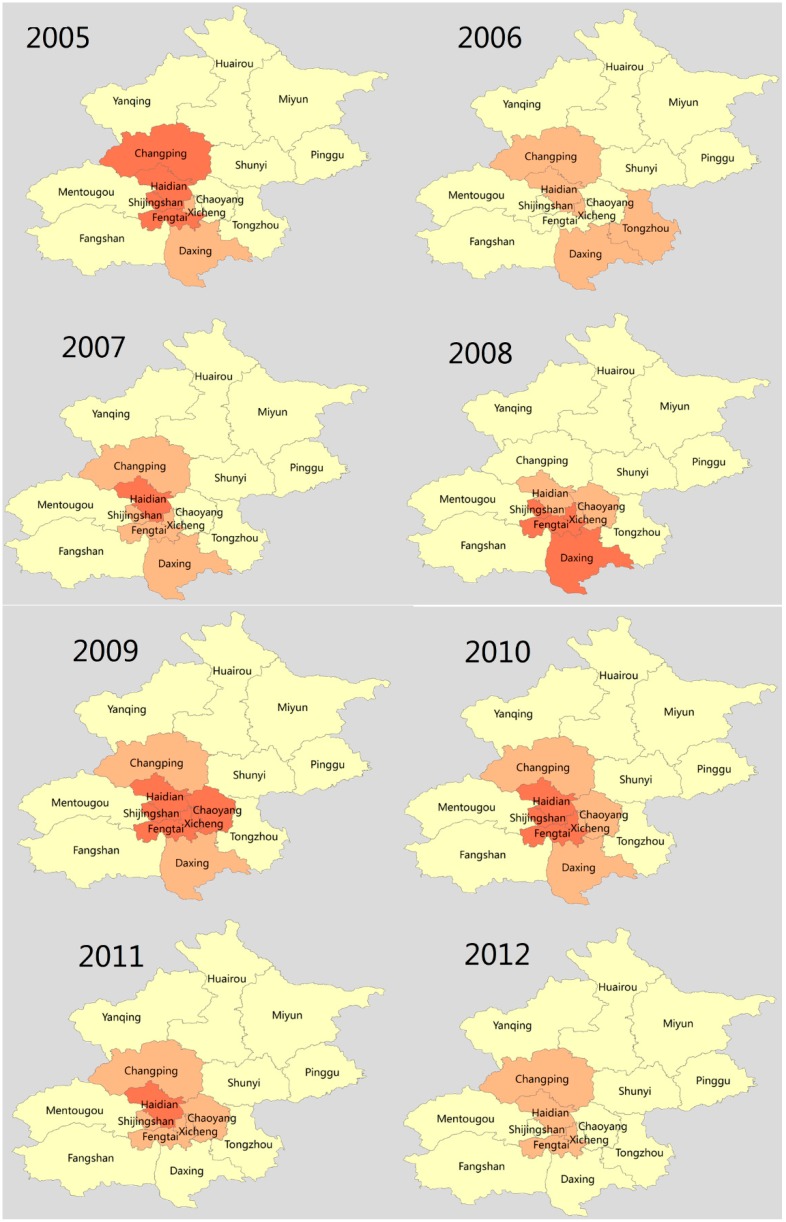

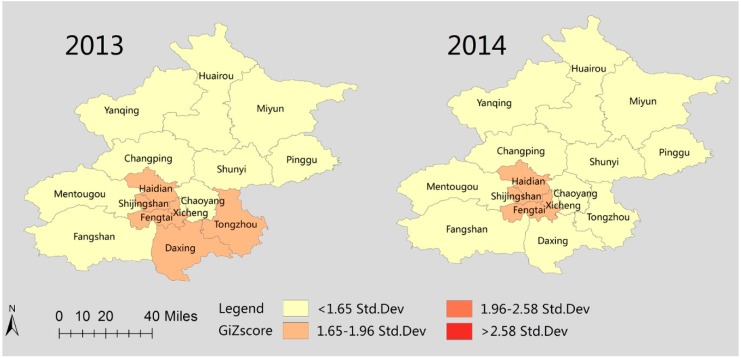
Hotspot clusters of scarlet fever incidence in Beijing, 2005–2014.

Figure 3 demonstrates the hotspots of scarlet fever incidence at the district level in Beijing, during the study period. The score of Giz >2.58 reflects the presence of disease aggregation, with a probability of 99%, using Getis-Ord Gi* statistic. The locations and size of hotspots varied in each year. The main hotspots of scarlet fever were identified in urban districts, such as Xicheng, Haidian, Fengtai, and Shijingshan, and two rural districts (Doxing and Changping). Notably, no hotspot areas were detected in the rural districts of Beijing, except for Doxing and Changping districts (Figure 3).

### 3.5. Spatial Clusters of Scarlet Fever Disease

**Figure 4:**
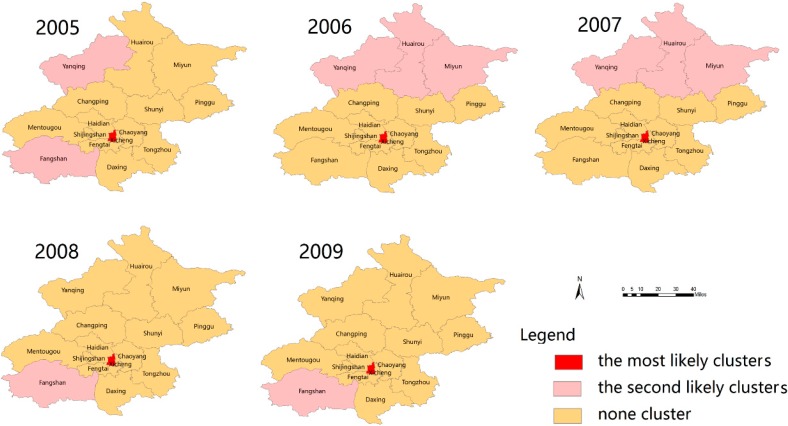

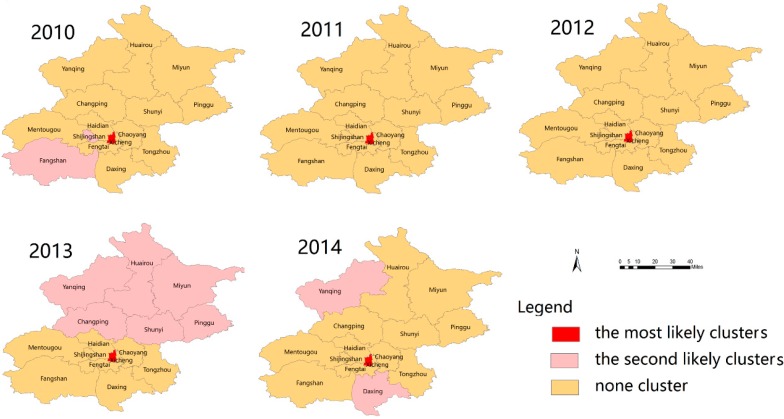
The most likely clusters and the secondary clusters of scarlet fever incidence in Beijing were detected using the Purely Spatial Poisson model, 2005–2014. Red indicates the most likely cluster, slightly red indicates secondary clusters and light red indicates no clusters in the study areas.

Using the discrete Poisson model, we detected the statistically significant, most likely spatial clusters, and several secondary clusters of scarlet fever in Beijing by setting a maximum spatial cluster size of 50% of the total population (Figure 4). In this study, the location and size of these clusters varied over the study period. The most likely cluster was a single area detected in the Xicheng district during the entire study period (2005–2014); the Xicheng district is an urban district of Beijing. Several secondary clusters were detected inconsistently in each year (Figure 4). Table S1 summarizes the location, size, annual cases per 10^5^, likelihood ratio, and relative risk, with a *p*-value of the most likely spatial clusters in Beijing (2005–2014).

### 3.6. Space-Time Clusters

**Figure 5:**
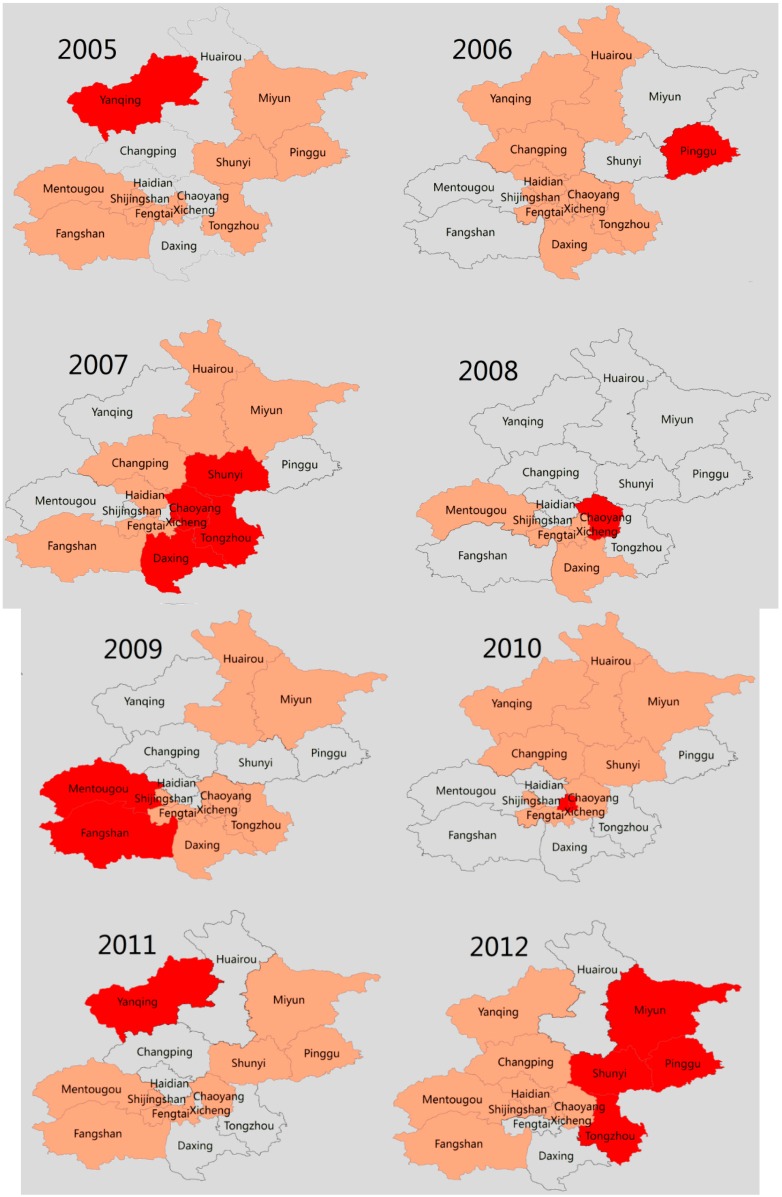

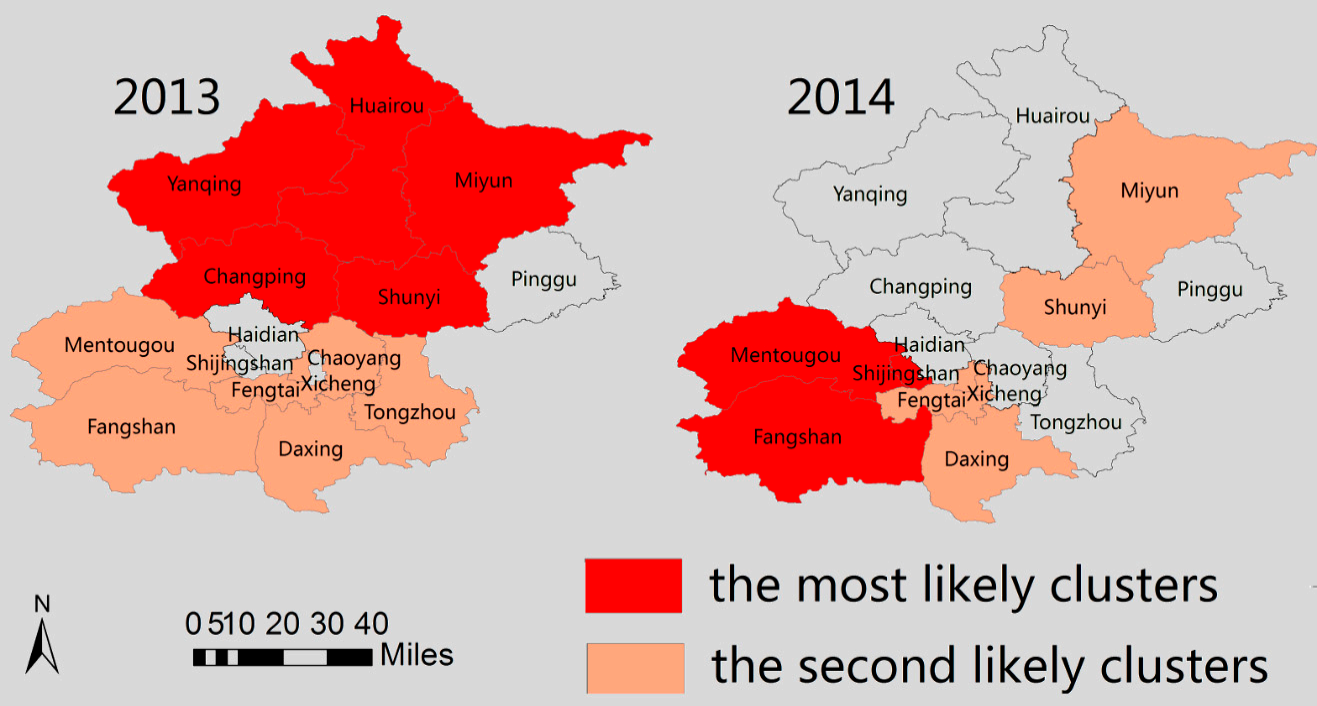
The most likely clusters and the secondary clusters of scarlet fever incidence in Beijing using a space-time permutation model, 2005–2014.

Using a space-time permutation model, we identified the statistically significant monthly spatiotemporal clusters with a high incidence of scarlet fever from 2005 to 2014 (Table 4, Figure 5). The incident of scarlet fever had space-time heterogeneity in the Beijing region during the study period (Figure 5).

**Table 4:** The most likely high-risk clusters of scarlet fever incidence detected using a space-time permutation model (setting 50% as the maximum cluster size) in Beijing, 2005–2011.

Scan Year	Cluster Time Frame	Center(Latitude, Longitude)/Radius (km)	Cluster Districts	Relative Risk	*p*-Value
2005	1 January to 30 April	116.156 E, 40.537 N/0	1	1.90	0.014
2006	1 May to 31 August	117.135 E, 40.205 N/0	1	1.97	0.000049
2007	1–31 January	116.728 E, 39.801 N/35	4	1.55	0.012
2008	1 January to 29 February	116.507 E, 39.949 N/0	1	1.82	0.00029
2009	1 January to 28 February	39.99 N, 115.78 E/30.98	2	2.22	0.109
2010	1–31 December	116.410 E, 39.911 N/4.34	2	1.28	0.081
2011	1 September to 31 December	116.156 E, 40.537 N/0	1	2.97	<0.001
2012	1 April to 30 June	117.135 E, 40.205 N/56.68	4	1.49	0.0000014
2013	1 July to 30 September	116.579 E, 40.628 N/55.86	5	1.87	0.017
2014	1 September to 30 November	39.991 N, 115.787 E/33.31	3	1.43	0.107

Note: “Scan year” indicates the boundary of time points input into the scanning analysis, and the “Cluster time frame” indicates the boundary of time points identified by the scanning analysis.

The most likely clusters detected varied in each study year in the study area. Five clusters were detected in 2013 in the following rural districts: Huairou, Miyun, Yanqing, Shunyi, and Changping), and the cluster time ranged from 1 July to 30 September; the cluster centre was (116.57° E, 40.62° N), and the radius was 55.86 Km, with a relative risk (RR) value of 1.87 (*p* > 0.017), followed by four clusters in 2007 (Tongzhou, Chaoying, Dongcheng, Shunyi districts) and 2012 (Pinggu, Shunyi, Miyun and Tongzhou districts). In addition, we identified three most likely clusters in 2014 and two clusters in 2009 and 2010; the rest of the year, we detected a single cluster (Figure 5, Table 4). There were several secondary clusters identified apart from the most likely clusters (Figure 5).

## 4. Discussion

We have provided geographical clusters of scarlet fever incidence in Beijing through exploratory disease mapping, spatial autocorrelation analysis and hotspot analysis. We detected that the incidence of scarlet fever varied greatly over time, and the Beijing region experienced the highest number of reported cases (6566) in 2011. The annual incidence of scarlet fever was persistently greater among males than among females, particularly among nursery, kindergarten and pre-primary school children, who were found to be more vulnerable to scarlet fever during 2005–2014. The incidence of scarlet fever was high among children 3–8 years of age (83.8%) during epidemic and non-epidemic years in Beijing (Table 2). A study in Hefei, China, also reported similar results from 2004 to 2008 [28]. Likewise, a previous study by Chen, *et al.* reported that the maximum incidence (85.9%) was among children 4–9 years of age. Peng *et al.* [17] reported similar results in Beijing from 2006 to 2011, with a higher incidence among males than among females. Additionally, several studies in Hong Kong [22] and Poland [20] found a higher incidence among children 4–7 years of age. The WHO and Public Health, UK, stated that the scarlet fever infection is most common among children 5–15 years old [2,56].

Our study revealed that the incidence of scarlet fever seemed to be slightly increased from 2005 to 2008; however, it was notably lower in 2009 and 2010 (6.76 cases/100,000 population and 8.50 cases/100,000 population, respectively). Unexpectedly, scarlet fever broke out in 2011 with 32.03 cases/100,000 population in Beijing, which is consistent with the findings of Peng *et al.* [17], who reported 7.0 to 14.3 cases/100,000 populations from 2006 to 2010 and 31.4 cases/100,000 populations in 2011 in the same study area. Interestingly, the Luk, *et al.* study [22] reported that the incidence of scarlet fever showed a decreasing trend in Hong Kong from 2008 to 2010, whereas another study suggested that the incidence of scarlet fever had decreased prior to the 2008 Olympic Games in Beijing [26]. However, our finding showed that the scarlet fever incidence demonstrated an increasing trend from 2005 (14.7) until 2007 (14.27), and then dropped in 2008 (10.15 cases/100,000 population). Furthermore, the study of You, *et al.* [25] stated that the incidence of scarlet fever was at a lower level in the eight years prior to the scarlet fever incidence in 2011. Indeed, 2011 was an epidemic year of scarlet fever in Beijing, Shanghai, Hong Kong, Macau and Taiwan [2].

The autocorrelation analysis of Global Moran’s *I* value demonstrated that the spatial distribution of scarlet fever became more uneven (Table 3). However, Anselin Local Moran’s *I* analysis revealed that the circular area with a high-high positive spatial association with the incidence of scarlet fever was located around the urban districts (Xicheng, Chaoyang, and Fengtai) in Beijing (Figure S1). In addition, the Getis-Ord Gi* statistic indicated several hotspot clusters in urban districts (Haidian, Shijingshan, Fengtai, Xicheng and Chaoyang) and few in the rural Changping and Daxing districts*)* in densely populated areas, supporting a positive relationship between population density and an increased risk of scarlet fever exposure.

The temporal analysis showed that during the epidemic year 2011 and throughout 2005 to 2014, scarlet fever cases peaked twice yearly. The first peak occurred in May (at the end of spring), and a smaller, second peak was observed in December (early winter) in Beijing. More clearly, the peak began in March and reached its highest level in May, and a second smaller peak began in October and reached its highest level in December in each year. A consistent finding was reported by Peng *et al.* [17] and Chen *et al.* [29] in Beijing and Shanghai, respectively. Some studies have reported that the incidence peak was observed in March in Hefei*,* China, [28] and in the United Kingdom [21], and in June in Hong Kong [22]. It has been widely known that scarlet fever primarily occurs in the winter/spring seasons [2,56,57]. Notably, the seasonal peaks varied in different regions. These differences might be partially attributed to climate, geographic distribution, and social factors. This persuasive evidence indicates that scarlet fever transmits more easily in spring and winter compared to the other season, which is consistent with previous studies, as well with WHO data [2].

Furthermore, spatial cluster scanning results showed that the centre of the most likely cluster was detected in Xicheng district in the urban area during our entire study period and that the secondary cluster detected was diverse in each district in the different time periods. The highest relative risk (10.12), with annual cases of 228.2/100,000 population, a likelihood ratio of 2850.73, and a *p-*value < 0.001, was observed in 2011 in Beijing, which was an epidemic year for scarlet fever [2]. Additionally, the most likely clusters, along with secondary cluster locations, were found in densely populated districts, with major transmission spots, such as markets, railways and highways, which suggest that scarlet fever might be transmitted through railways and highways from one place to another. This result also reminds us that prevention and control measures for scarlet fever should focus more on the most likely and secondary cluster areas, including neighbouring districts, to maximize the cost-effectiveness of prevention efforts.

Moreover, our study found the most likely space-time clusters in diverse patterns in Beijing, including time frame, cluster centre, relative risk with and *p* value. The most likely clusters were observed in rural districts (Huairou, Yanqing, Miyun, Shunyi, Pinggu, Changping) and in urban districts (Xicheng, Chaoyang and Shijingshan*),* and several secondary clusters were found in urban and rural districts (Figure 5, Table 4). Information about high risk clustered and secondary clustered districts, including neighbouring districts, might be helpful in preventing disease infection and to manage public health planning and resource allocation in advance, to minimize costs and reduce complications.

There are several limitations in this study. First, we used data from reported cases of scarlet fever (*i.e.*, confirmed cases using diagnosis criteria), however, we were unable to explore the characteristics of group A streptococcus GAS *emm typing* stain *(*e.g., *emm1, emm3 emm4, emm6, emm12, or emm22),* according to the protocol described by the US CDC due to unavailable data for such information. Second, scarlet fever has been listed as a notifiable infectious disease in China since 2004, and we could not assure the accuracy of the relatively new surveillance monitoring system because some mild cases might be missed or might not be reported (they might utilize home remedies). In this context, the patient information might be lost. Third, this study used circular spatial scanned statistic, which does not allow for irregular geographic shapes. Likewise, the small clusters in the early years could not be identified when data from all 119 months were used. We treated this limitation by scanning each year independently, which has the disadvantage of losing information from each year in the further clusters. Fourth, environmental factors, such as temperature, humidity, rainfall, precipitation and wind pressure, could influence scarlet fever infection, but such data have been available since only 2012 in Beijing. Therefore, we were unable to include these data in our study.

## 5. Conclusions

In conclusion, our study provides a good understanding of the spatiotemporal patterns of scarlet fever in Beijing from 2005 to 2014. The at-risk population was mostly distributed in urban areas and highly and densely populated districts, which indicates a positive relationship between population density and increased risk of scarlet fever exposure. Children under 15 years of age were more vulnerable to scarlet fever infection. With the help of spatiotemporal framework analyses, disinfection should be strengthened and management should focus on the scarlet fever cluster areas and cluster times (*i.e.*, in the urban districts of Beijing). This focus may help control the scarlet fever epidemic more effectively and reduce its harm to children.

## Supplementary Materials

The following are available online at www.mdpi.com/1660-4601/13/1/131/s1, Table S1: The most likely clusters of scarlet fever disease in Beijing, 2005–2014, using purely spatial analysis of the discrete Poisson model (setting 50% as the maximum cluster size). Figure S1. Local Moran’s *I* analysis of scarlet fever in Beijing, 2005–2014.
