# Supplementary material for: Spatiotemporal Pattern Analysis of Scarlet Fever Incidence in Beijing, China, 2005–2014
Source: Int J Environ Res Public Health. 2016 Jan 15;13(1):131. doi: 10.3390/ijerph13010131 (PMC4730522; doi:10.3390/ijerph13010131)
Supplement: Supplementary file 1 [file ijerph-13-00131-s001.pdf]

Supplementary Materials: Spatiotemporal Pattern Analysis of Scarlet Fever Incidence in Beijing, China, 2005–2014

Gehendra Mahara, Chao Wang, Da Huo, Qin Xu, Fangfang Huang, Lixin Tao, Jin Guo, Kai Cao, Liu Long, Jagadish K. Chhetri, Qi Gao, Wei Wang, Quanyi Wang and Xiuhua Guo

Table S1. The most likely clusters of scarlet fever disease in Beijing, 2005–2014, using purely spatial analysis of the discrete Poisson model (setting 50% as the maximum cluster size).

Scan Year	Number of Cluster	Annual Cases (/10^5)	LLR	RR	*p*-Value
2005	1	50.8	423.20	5.66	<0.001
2006	1	62.0	574.80	6.46	<0.001
2007	1	62.0	574.80	6.46	<0.001
2008	1	48.6	476.13	6.92	<0.001
2009	1	31.5	281.33	6.17	<0.001
2010	1	49.5	527.91	7.84	<0.001
2011	1	228.2	2850.73	10.12	<0.001
2012	1	103.8	1152.66	8.29	<0.001
2013	1	82.0	1087.13	11.28	<0.001
2014	1	128.8	1748.29	11.78	<0.001

LLR = Log likelihood Ratio; RR = Relative Risk.

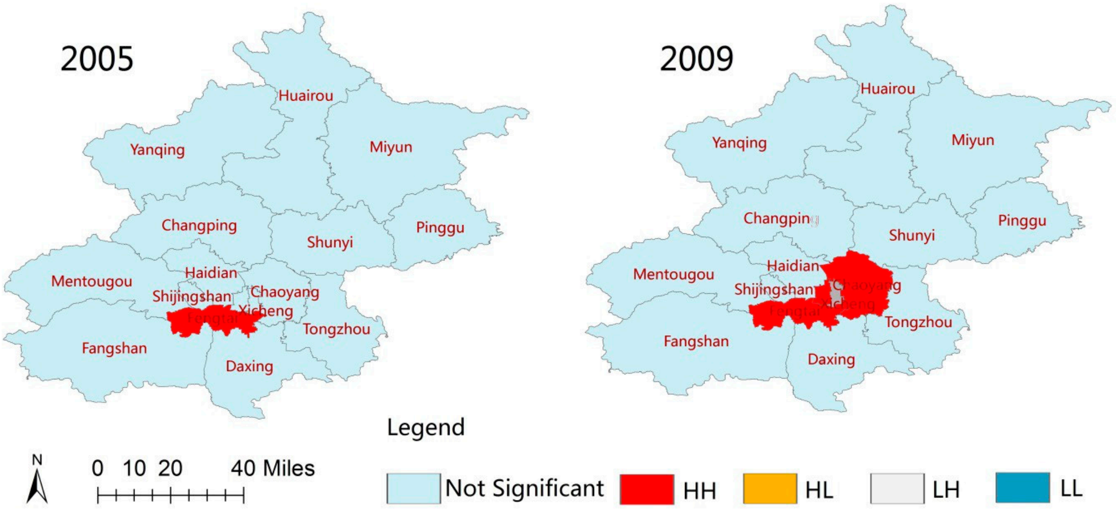

Figure S1. Local Moran's *I* analysis of scarlet fever in Beijing, 2005–2014.
